# The urgent need to end sponsorship of healthcare professional associations by the commercial milk formula industry

**DOI:** 10.1016/j.fhj.2025.100265

**Published:** 2025-06-30

**Authors:** Tony Waterston, Adriano Cattaneo, Nicole Bando, Jamie Errico, Robert Boyle, Sarah Brennan, Lori Lake, Melissa Mialon, Laurence Grummer-Strawn, HASFF (Health Advocates for Sponsorship- Free Feeding)

**Affiliations:** a Population Health Sciences, University of Newcastle upon Tyne, Newcastle upon Tyne, UK; b IBFAN Italy, Trieste, Italy; c NEST Family Clinic, Elsternwick Victoria, Australia; d Department of Nutrition, Dietetics and Food, School of Clinical Sciences, Monash University, Melbourne, Australia; e School of Public Health, University College, London, United Kingdom; f School of Medicine, College of Medicine, Nursing and Health Sciences, University of Galway, University Road, Galway, Ireland; g Children’s Institute, University of Cape Town, Rondebosch, South Africa; h Université Paris Cité, Paris, France; i Food & Nutrition Action in Health Systems, World Health Organization, Geneva, Switzerland

**Keywords:** Sponsorship, Conflict of interests, Infant feeding, Commercial determinants of health, Health professional associations, International code

## Abstract

Sponsorship of healthcare professional associations (HCPAs) by the commercial milk formula (CMF) industry violates the International Code of Marketing of Breastmilk Substitutes. The financial ties and conflicts of interests brought about by such sponsorship distort education, research and practices in ways which undermine breastfeeding and compromise maternal and child health. The Health Advocates for Sponsorship-Free Feeding (HASFF) working group, convened by the World Health Organization, aims to address these challenges and has developed a range of tools and resources to support and encourage other HCPAs to end sponsorship from the CMF industry.

## Introduction

It is 44 years since the adoption of the International Code of Marketing of Breastmilk Substitutes (The Code) by the World Health Organization (WHO), intended to curb the aggressive and inappropriate marketing of commercial milk formula (CMF), which was damaging the health of infants and young children globally.^1^ Despite decades of advocacy, many World Health Assembly (WHA) resolutions and an FAQ on the Code in 2017,^2^ the consequences of intrusive marketing remain more critical than in 1981 and the pervasive impact of the commercial sector has extended across the whole field of infant feeding.^3^ This article aims to encourage health professional associations to take action to end industry sponsorship and join our growing coalition of health professionals advocating for sponsorship-free infant and young child feeding.

## Using the health sector to market CMF

‘Medical marketing’ or the use of the health sector – including hospitals, health centres, health staff and healthcare professional associations (HCPAs) – has been a core marketing strategy of the CMF industry from its inception.^4^ The ongoing, problematic nature of CMF marketing practices was highlighted in a special series of *The Lancet* in February 2023.^5^ One of the *Lancet* papers stressed that ‘CMF marketing is a multifaceted, sophisticated, well resourced, and powerful system of influence that generates demand and sales of its products at the expense of the health and rights of families, women, and children. Digital platforms and use of individual data for personalised and targeted marketing have substantially enhanced the reach and influence of this system.’^3^

Despite the key role that HCPAs play in supporting CMF marketing, health professionals are often unaware of the ways in which these financial ties are damaging to breastfeeding or child health.^3^^,^^6^^,^^7^ Our particular concern is the sponsorship of HCPAs by the CMF industry – something that affects most national paediatric associations,^8^ among other specialties. The impact of such sponsorship is to distort education, research and clinical practice in ways that favour earlier, more prolonged formula feeding with more expensive products. Industry support of HCPAs affects individual priorities, biases policies and distorts decision-making.^9^^,^^10^

## Arguments used to justify sponsorship

When HCPAs are challenged over their practice of accepting sponsorship, a variety of arguments are used to defend the practice.^11^ These include the proposition that there is a lack of evidence that sponsorship affects decision-making or child health and that the impact is different in different countries. Evidence, however, shows that sponsorship of HCPAs is an explicit marketing strategy of the CMF industry which invests in healthcare professionals (given their central role as trusted health providers) in order to increase its profits by increasing the number of children fed with CMF; and that as sales of CMF increase, breastfeeding rates decline.^12^ When restrictions on marketing are introduced in national law, as aligned with the Code, then breastfeeding rates increase.^13^

Some HCPAs respond that CMF is not necessarily damaging to infant health. This argument runs counter to overwhelming evidence that not breastfeeding increases children’s risk for a wide range of adverse health outcomes–notably gastroenteritis, respiratory infections and necrotising enterocolitis.^14^ Breastfeeding is protective against development of cardiometabolic disease, including overweight, obesity and type 2 diabetes^15^ and it improves maternal heath in disparate ways, such as reduced risk of ovarian and breast cancers, type 2 diabetes.^14^ There are indeed infants who may need to be formula fed, ie when breastfeeding is not available because the mother is absent or ill. The problem is that marketing expands the use of CMF to those infants who do not require it and could have been breastfed,^3^ or fed with donated human milk.

Another argument is that infants and children deserve access to safe products that the industry can provide.^11^ This is certainly true, but sponsorship of HCPAs does not affect access to products, and HCPAs are not responsible for ensuring the safety of CMF. All CMF need to be nutritionally adequate and safe; and it is the responsibility of manufacturers to abide by government regulations.

It is also suggested that paediatricians and other professionals need access to accurate information about products and need to have a close relationship with CMF companies for this reason.^11^ Importantly, professionals need a source of accurate information which is independent of commercial sources to avoid any possible bias^16^^,^^17^ and they need to be free of conflict of interest arising from fiscal inducements.

Finally, supporters of sponsorship state that HCPAs rely on industry funding for their educational programmes, both for trainees and for lifelong learning. However, there are many successful associations that find other sources of funding.^18^ While CMF funding may make organisation of meetings easier, it detracts from HCPAs’ primary mission to foster an environment that promotes child health. There is evidence that there is always a risk of bias in prescription of CMF following interactions between health professionals and industry.^3^ Similarly, there is abundant evidence that interactions between physicians and pharmaceutical representatives influence drug prescribing practice.^19^

## WHA resolutions call for an end to sponsorship

For these reasons, the WHA has called on HCPAs to develop policies prohibiting CMF sponsorship (Resolution WHA 69.9 of 13 May 2016).^20^ The associated guidance^21^ states that CMF ‘companies, or their representatives, should not … sponsor meetings of health professionals and scientific meetings’, and that ‘health workers, health systems, health professional associations and nongovernmental organizations should not … allow such companies to sponsor meetings of health professionals and scientific meetings’. According to the WHO, therefore, the responsibility for ending CMF sponsorship falls on both CMF companies and health professionals. Furthermore, as WHA recommendations are addressed to governments, it is their role to implement legislation on CMF sponsorship, from regulations to bans with adoption of the Code into national law. Until now, there has been only partial uptake of WHA recommendations, with evasion of the recommendations by large numbers of HCPAs.^3^ The present initiative was established to bring together HCPAs that have ended sponsorship to motivate and inspire other associations to follow their lead.

## The formation of HASFF

To advance implementation of this recommendation, WHO convened a working group called Health Advocates for Sponsorship-Free Feeding (HASFF).^1^ This group of health professionals drawn from medicine, nursing, midwifery and other organisations brings together a multiprofessional team of 25 people from five continents. The goal of the working group is to progressively increase the number of HCPAs that end sponsorship by the CMF industry. The group meets online once a month (35 regular meetings so far) to discuss and plan actions that will then be carried out by smaller working groups.

## Work carried out by HASFF

Action up to now includes:

• Planning of a 2022 global meeting of associations that have ended sponsorship to act as exemplars and disseminate their experience internationally
• The publication of a paper in *BMJ Paediatrics Open* examining and refuting the arguments made in favour of sponsorship^16^
• A letter to *The Lancet*, calling ‘upon all HCPAs to bring an end to sponsorship relationships with companies that market breastmilk substitutes’^22^
• Compilation of case studies of associations that have ended sponsorship by CMF companies^18^
• Development of a model policy for HCPAs on CMF sponsorship^23^
• Publication of a guide to alternative sources of funding for hosting sponsorship-free HCPAs events^24^
• Collation of a searchable repository of papers and international guidance on sponsorship of HCPAs and conflict of interest has also been developed and will soon be made available.

Currently, HASFF is developing a survey to assess the current situation of CMF sponsorship of HCPAs which could be used to monitor progress. A Community of Practice, linked to a new HASFF website, will soon be established which will allow and encourage discussion among health professionals and advocates of breastfeeding globally, on means of ending the corrupting influence of formula (and infant food) marketing throughout the health sector.

## Need for healthcare professional education

Changing the culture of HCPAs towards a resistance to commercial sponsorship requires the inclusion of modules on the commercial determinants of health in basic and further education curricula of health professionals. Within these modules, themes taught need to encompass WHO policies on infant feeding, the Code, sponsorship and conflict of interest, marketing of commercial products through the health system, and patterns of normal infant feeding behaviour and support for breastfeeding mothers.^9^ New teaching programmes that bring together medics, nurses, midwives, nutritionists and other HCPAs in the same room are needed, to facilitate cross-discipline collaboration and exchange of insights. A no-advertising of CMF policy for medical journals, in particular those affiliated with HCPAs,^25^ could further reinforce the above mentioned educational initiatives.

## Call for action

Early life feeding practices are critical influences on the growth, development and survival of infants and young children, and also impact the health of their mothers.^14^ Building on the impetus of these recent policy and advocacy developments, we call on health professionals and their associations across the world to assist us in building a coalition of support for sponsorship-free infant and young child feeding.

## Funding

This research did not receive any specific grant from funding agencies in the public, commercial or not-for-profit sectors.

## CRediT authorship contribution statement

**Tony Waterston:** Writing – review & editing, Writing – original draft, Conceptualization. **Adriano Cattaneo:** Writing – original draft, Conceptualization. **Nicole Bando:** Writing – review & editing, Conceptualization. **Jamie Errico:** Writing – review & editing. **Robert Boyle:** Writing – review & editing, Validation, Data curation. **Sarah Brennan:** Writing – review & editing. **Lori Lake:** Writing – review & editing. **Melissa Mialon:** Writing – review & editing, Methodology. **Laurence Grummer-Strawn:** Writing – review & editing, Methodology, Data curation, Conceptualization.

## Declaration of competing interest

The authors declare that they have no known competing financial interests or personal relationships that could have appeared to influence the work reported in this paper.

## Footnotes

1 Formerly called Practitioners in Health Against Sponsorship by the Formula Industry (PHASFI).
